# Supplementary material for: Accuracy of intraocular lens power calculation formulas using a swept-source optical biometer
Source: PLoS One. 2020 Jan 14;15(1):e0227638. doi: 10.1371/journal.pone.0227638 (PMC6959581; doi:10.1371/journal.pone.0227638)
Supplement: Attachment — Submitted filename: Respond to reviewers_191128.docx [file pone.0227638.s002.docx]

November 28, 2019

Editor in Chief

PLOS ONE

Thank you very much for your letter regarding our revised manuscript. We revised the manuscript as recommended by the editor and reviewer. Each of the coauthors has seen and agrees with each of the changes made to the manuscript. Our responses to the comments of the editor and reviewer, including the changes made in the manuscript, are provided in a point-by-point manner in the following pages.

We hope that you will find the manuscript suitable for publication in your esteemed journal.

Yours sincerely,

Ji Won Jung, MD, PhD

Department of Ophthalmology, Inha University Hospital, 7-206, 3-ga,

Shinheung-dong, Jung-gu, Incheon 400-711, South Korea

Tel.: 82-32-890-2400, Fax: 82-32-890-2403, E-mail: panch325@gmail.com

Reviewer Comments:

Please use the space provided to explain your answers to the questions above. You may also include additional comments for the author, including concerns about dual publication, research ethics, or publication ethics. (Please upload your review as an attachment if it exceeds 20,000 characters)

Reviewer #1: Just a minor comment: in table 1, medium axial length is still defined as between 22.5 and 26 mm. Please correct 22.5 and change it into 22

🡪 Thank you for your comment. We corrected it.

Reviewer #2: Good results that support the conclusion. Reasonable statistical analysis. Correct terminology used. The ideas are clearly expressed in English. A bigger sample size is suggested in future research.

🡪 Thank you for your comment. We added the need of bigger sample size in future research in Discussion section.

Reviewer #4: We have confirmed the corrections in the manuscript.

Please indicate the minimum and maximum values for the box & whisker plots.

🡪 Thank you for your comment. We added the minimum and maximum values in Fig2.
